# Auxiliary use of ChatGPT in surgical diagnosis and treatment – correspondence

**DOI:** 10.1097/JS9.0000000000000818

**Published:** 2023-10-06

**Authors:** Qing-xin Yu, De-chao Feng, Rui-cheng Wu, Deng-xiong Li

**Affiliations:** a Department of Pathology, Ningbo Clinical Pathology Diagnosis Center, Ningbo City, Zhejiang Province; b Department of Urology, Institute of Urology, West China Hospital, Sichuan University, Chengdu, Sichuan Province, People’s Republic of China

*Dear Editor,*

As pivotal constituents of artificial intelligence (AI), large language models (LLMs) have captured considerable research interest due to their transformative potential. Notably, Yang and Au^1^ have recently elucidated the prospective role of ChatGPT as a surgical tool, shedding light on comprehensive challenges in clinical application. We extend our commendations for their exemplary contributions. However, we believe there exist several prospective areas for enhancement within this domain. Consequently, we seek to initiate a discourse on these issues at the preliminary phase of ChatGPT’s integration into surgical practice.

ChatGPT undergoes significant version updates, as seen in instances like ChatGPT 3.5 and ChatGPT 4, with each version’s nomenclature reflecting its enhanced capabilities. The specific version number serves as a functional indicator for ChatGPT. In the study conducted by Yang and Au, this distinction was not explicitly articulated, potentially introducing ambiguity for both their findings and readers. For instance, the authors suggested that ChatGPT might provide personalized and in-depth responses, while Google was better suited for retrieving broad information. This characterization aligns with ChatGPT 3.5, which was updated only until 2021^2^. However, ChatGPT 4 has the capacity to access the web for data retrieval, rendering the suggestion somewhat ambiguous. Consequently, we propose that specifying the ChatGPT version used would enhance the overall quality and clarity of their study.

It is imperative to acknowledge that, due to current limitations, neither ChatGPT 3.5 nor ChatGPT 4 is capable of performing surgical procedures^3^. Moreover, the formulation of precise surgical plans, encompassing detailed steps and techniques, has been recognized as a challenge^4^. Erroneous results in this regard carry the potential to inflict significant harm on patients. In this context, ChatGPT holds promise in offering valuable insights and novel research directions, optimizing time management, and expanding the scope of our knowledge^5^. Therefore, we concur with the notion that ChatGPT 3.5/4 can be a collaborator who provides perioperative recommendations and up-to-date research insights during surgical procedures rather than directly engaging in specific surgical actions. A notable Japanese study has introduced an AI model capable of autonomously assessing surgical skills, representing a potential initial step toward ChatGPT’s involvement in surgical procedures^6^. ChatGPT could potentially advise surgeons on specific actions based on extensive high-scoring surgical video data; although this approach may require substantial time investments, it could yield results more closely aligned with clinical practice. In light of these considerations, it is judicious to perceive ChatGPT as a valuable ally in our medical endeavors, playing a pivotal role as a partner complementing clinical surgical practice rather than replacing surgeons.

Collectively, the efforts of Yang and Au produce novel and clinically substantial findings. These insights have the capacity to stimulate advancements in this field, enriching the trajectory of this discourse and offering an alternative perspective for forthcoming investigations.

## Ethical approval

Not applicable.

## Consent

Not applicable.

## Sources of funding

No funding.

## Author contribution

Q.-x.Y., D.-c.F., R.-c.W., and D.-x.L.: collected and reviewed the literature and designed the writing; D.-x.L.: revised the manuscript. All authors read and approved the final manuscript.

## Conflicts of interest disclosure

All the authors declare that there are no relevant conflicts of interest to disclose.

## Research registration unique identifying number (UIN)

Not applicable.

## Guarantor

Qing-xin Yu, De-chao Feng, Rui-cheng Wu, and Deng-xiong Li.

## Data availability statement

Not applicable.

## Provenance and peer review

Not commissioned.
